# Advances in using Internet searches to track dengue

**DOI:** 10.1371/journal.pcbi.1005607

**Published:** 2017-07-20

**Authors:** Shihao Yang, Samuel C. Kou, Fred Lu, John S. Brownstein, Nicholas Brooke, Mauricio Santillana

**Affiliations:** 1 Department of Statistics, Harvard University, Cambridge, MA, USA; 2 Computational Health Informatics Program, Boston Children’s Hospital, Boston, MA, USA; 3 Harvard Medical School, Boston, MA, USA; 4 The Synergist, Brussels, Belgium; Ecole Polytechnique Federale de Lausanne, SWITZERLAND

## Abstract

Dengue is a mosquito-borne disease that threatens over half of the world’s population. Despite being endemic to more than 100 countries, government-led efforts and tools for timely identification and tracking of new infections are still lacking in many affected areas. Multiple methodologies that leverage the use of Internet-based data sources have been proposed as a way to complement dengue surveillance efforts. Among these, dengue-related Google search trends have been shown to correlate with dengue activity. We extend a methodological framework, initially proposed and validated for flu surveillance, to produce near real-time estimates of dengue cases in five countries/states: Mexico, Brazil, Thailand, Singapore and Taiwan. Our result shows that our modeling framework can be used to improve the tracking of dengue activity in multiple locations around the world.

## Author summary

As communicable diseases spread in our societies, people frequently turn to the Internet to search for medical information. In recent years, multiple research teams have investigated how to utilize Internet users’ search activity to track infectious diseases around our planet. In this article, we show that a methodology, originally developed to track flu in the US, can be extended to improve dengue surveillance in multiple countries/states where dengue has been observed in the last several years. Our result suggests that our methodology performs best in dengue-endemic areas with high number of yearly cases and with sustained seasonal incidence.

## Introduction

Dengue fever poses a growing health and economic problem throughout the tropical and sub-tropical world. Dengue is today one of the fastest-growing and most important mosquito-borne viral diseases in the world, with an estimated 390 million infections each year and threatening an estimated 3.9 billion people in 128 countries [1]. Infection often causes high fever and joint pain, and severe cases can lead to hemorrhage, shock and death. Dengue epidemics strain health services and lead to massive economic losses.

Dengue transmission is subject to complex environmental factors influencing the *Aedes aegypti* and *albopictus* mosquitoes which spread the disease. A mosquito is able to transmit dengue within a few weeks of contracting the virus, and a person bitten by such a mosquito will usually fall ill within a week, with symptoms lasting for up to 10 days afterward [2, 3]. There is a 5-day window when another mosquito can pick up the virus from an infected person [3]. The time scale of these transmission dynamics lends itself to tracking patterns of infection at a weekly or monthly level. Seasonal conditions such as temperature and precipitation can affect mosquito feeding rate, development, and lifespan, contributing to annual seasonality in observed dengue cases [4–8]. Other factors affecting the local or regional level include human population density and mobility, mosquito control efforts, and the distribution of the four dengue virus serotypes, adding complications to efforts to model transmission dynamics [9, 10].

Dengue mortality and morbidity both need to be addressed to reduce this heavy burden. The World Health Organization has called for better early case detection among other tactics to reduce dengue mortality, and for the reduction of dengue morbidity through coordinating epidemiological and entomological surveillance. Timely identification of outbreaks can inform and help preventative measures to lower infection rates, including mosquito population control and providing supplies such as screens and nets for mosquito bite prevention. Thorough, data-informed implementations of these vector control methods have been found effective in reducing case counts in many locations, but require sustainable investment to prevent resurgence [3, 11], highlighting the need for accurate and timely dengue surveillance tools.

However, such a comprehensive, effective and reliable disease surveillance system for dengue is not yet available. Governments traditionally rely on hospital-based reporting, a method that is often lagged and limited with frequent post-hoc revisions, due to communication inefficiencies across local and national agencies and the time needed to aggregate information from the clinical to the state level [12, 13]. This lack of timely information limits the identification and optimization of effective interventions. Measurement difficulties are compounded by the fact that a majority of dengue cases are asymptomatic [14].

In this context, building an effective disease surveillance tool is essential to being able to identify and assess the severity of dengue outbreaks and to enable better assessment of the effectiveness of ongoing interventions. Such tools should provide accurate and consistent measures of regional or national infection levels, be updated in near real-time, and be immune to bureaucratic or resource-related delays. To improve accuracy, these tools should use and link together multiple sources of information, using both traditional and non-traditional sources.

Over the years, a broad range of traditional epidemiological methods have been proposed by research teams to fill this time gap of information by supplementing official case counts with now-cast estimation using dengue incidence data from previous seasons. Autoregressive models, such as Seasonal Autoregressive Integrated Moving Average (SARIMA) model, that take into account recent and seasonal patterns, have been shown to produce useful disease estimates, some including additional variables such as concurrent weather information [15–19]. Other studies have further examined climate-driven models, finding associations of seasonal and long-term weather patterns such as El Niño with dengue levels in various countries [5, 20–22]. In addition, various mechanistic models on the dynamics of dengue transmission have also been explored, with some recent promise [23]. A comprehensive survey of these methods are given in Andraud et al. [24]

In parallel and complementary to the aforementioned methodologies, the global spread of the Internet has opened up the opportunity to investigate whether users’ activity patterns on Internet search-engines and social media platforms may lead to reasonable estimates of dengue infection levels [25–27]. In theory, Internet search tracking is consistent, efficient, and reflects real-time population trends, giving it strong potential to supplement current epidemiological methods [13, 28]. Studies have previously demonstrated the feasibility of using Internet search data to track dengue case counts [25, 27]. Google Dengue Trends (GDT), launched in 2011, was one of the first tools to quantitatively track dengue activity in multiple regions throughout the world by leveraging the aggregate Google search patterns of millions of users [25]. Since its start, the methodology behind GDT has been updated to address flaws found in its sister effort, Google Flu Trends [29–37], before finally being discontinued in August 2015. An assessment of GDT in Mexico showed mixed prediction accuracy compared to official case counts, with strong correlation in some states [38].

Despite progress in the use of both dengue time-series information (time series approaches [15]) and real-time Internet searches for dengue tracking [25], an approach for accurate tracking of dengue by combining the respective strengths of each data source has not been documented to the best of our knowledge. We extend a methodology recently introduced in the flu surveillance literature to combine dengue-related Google searches with dengue case count time-series to track dengue activity. Specifically, we evaluate the performance of the ARGO model (AutoRegressive model with GOogle search queries as exogenous variables), as introduced in [35], in tracking dengue in five countries/states around the globe: Mexico, Brazil, Thailand, Singapore, and Taiwan. These countries were chosen to explore the applicability of our approach in a diverse set of ecological situations where dengue has been identified as an important local threat. Our contribution shows that the lessons learned to track influenza in data-rich environments, like the United States, can be used to develop methodologies to track an often-neglected tropical disease, dengue, in data-poor environments.

## Materials and methods

### Data

We used two kinds of data sets for our study: (a) historical dengue incidence from government-led health agencies and (b) Google search fractions of dengue-related queries, aggregated at the national-level.

#### Dengue time-series data

*Mexico*. Monthly-aggregated dengue case counts data from January 2001 to August 2015 were obtained from Mexico’s Department of Epidemiology. http://www.epidemiologia.salud.gob.mx/anuario/html/anuarios.html

*Brazil*. Monthly dengue case counts data from January 2001 to December 2012 were obtained from the old website of Brazil’s Ministry of Health (http://dtr2004.saude.gov.br/sinanweb/tabnet/dh?sinannet/dengue/bases/denguebrnet.def) on July 14, 2015. This site is no longer accessible, since the Ministry has moved to a new website (http://portalsaude.saude.gov.br/index.php/situacao-epidemiologica-dados-dengue), which now publishes weekly dengue data from 2014-present. This site contains annual dengue cases since 1990 but no longer has the historical monthly data. We confirmed that the annual totals match the sum of case counts over each year in our dataset.

*Thailand*. Monthly dengue case count data from January 2003 to August 2015 were obtained from the Bureau of Epidemiology, Thailand (http://www.boe.moph.go.th/boedb/surdata/disease.php?ds=66). New data are published in an annual document available on the site.

*Singapore*. Weekly dengue case counts from January 10, 2004 to August 29, 2015 were obtained from the Singapore Ministry of Health and were aggregated into months. https://www.moh.gov.sg/content/moh_web/home/statistics/infectiousDiseasesStatistics/weekly_infectiousdiseasesbulletin.html.

*Taiwan*. Weekly dengue case counts from January 3, 2009 to March 19, 2016 were obtained from the Taiwan Ministry of Health and Welfare and were aggregated into months. http://nidss.cdc.gov.tw/ch/SingleDisease.aspx?dc=1&dt=4&disease=061&position=1.

#### Online search volume data

Google search fractions for dengue-related queries were obtained from Google Trends (www.google.com/trends).

#### Online search term selection

While we initially intended to use Google Correlate (www.google.com/correlate), which is designed to identify search terms correlating highly with a given time series over a given country, we found this tool unreliable as many of the search terms returned were not related at all to dengue. Consequently, we used the Google Trends (www.google.com/trends) tool to identify the top ten queries most highly correlated with the term ‘dengue’ in each country, ignoring terms unrelated to dengue (one feature of Google Trends is that in addition to the trends of a specific term, it gives the top query terms that are most highly correlated with the specific term). The monthly aggregated search fractions of these terms were then downloaded within the time period of interest for each country. The query terms used for each country in this study were later verified by native speakers of each language and are presented in Table A in S1 Text.

### Methods

We used the multivariate linear regression modeling framework ARGO (AutoRegressive model with GOogle search queries as exogenous variables) [35], previously used to track flu incidence using flu-related Google searches, to combine information from historical dengue case counts and dengue-related Google search frequencies with the goal of estimating dengue activity one month ahead of the publication of official local health reports. ARGO uses a training set that consists of a two-year moving time window (immediately prior to the month of estimation) and an *L*_1_ regularization approach, to identify the best performing parsimonious model [39]. This moving window approach allows the model to constantly improve its predictive ability by capturing the changing relationship between Internet search behavior and dengue activity.

#### ARGO model formulation

Our ARGO model assumes that more dengue-related searches will be observed in times when more people are affected (either experiencing symptoms or hearing about someone who may have been infected) by the virus. This is formalized mathematically via a hidden Markov model in ARGO as explained in [35].

Let *y*_*t*_ = log(*c*_*t*_ + 1) be the *log*-transformed dengue case counts *c*_*t*_ at time *t*, and *X*_*k*,*t*_ the *log*-transformed Google search frequency of query term *k* at time *t*. Then

$$y_{t}=\mu_{y}+\sum_{j\in J}\;\alpha_{j}y_{t-j}+\sum_{k\in K}\;\beta_{k}X_{k,t}+\epsilon_{t},\;\epsilon_{t}\;\overset{iid}{∼}\;N\left(0,\sigma^{2}\right),$$ (1)

where *J* is the set of auto-regressive lags, *K* is the set of Google query terms, and ***X***_*t*_ can be thought of as the exogenous variables to time series {*y*_*t*_} as introduced in [35].

#### ARGO model parameter estimation

We take *J* = {1, …, 12} ∪ {24}, i.e., *J* consists of the most recent 12 months and the month exactly two years ago. Such choice of *J* captures the influence of short and mid-term yearly fluctuations, as well as long-term seasonality previously shown to have strong predictive power in dengue [15]. We take *K* = 10, corresponding to the top ten dengue-related search terms as described in the data subsection.

We impose *L*_1_ regularity for parameter estimation. In a given month, the goal is to find parameters *μ*_*y*_, ***α*** = {*α*_*j*_: *j* ∈ *J*}, and ***β*** = (*β*_1_, …, *β*_10_) that minimize

$$\sum_{t}\;{\left(y_{t}-\mu_{y}-\sum_{j\in J}\;\alpha_{j}y_{t-j}-\sum_{k=1}^{10}\;\beta_{k}X_{k,t}\right)}^{2}+\sum_{j\in J}\;\lambda_{\alpha_{j}}|\alpha_{j}|+\sum_{k=1}^{10}\;\lambda_{\beta_{k}}\left|\beta_{k}\right|$$ (2)

where λ_*α*_*j*__, λ_*β*_*k*__ are regularization hyper-parameters.

For a given time window, ARGO automatically selects the most relevant variables to generate an out-of-sample dengue activity estimate. This is achieved by zeroing out regression coefficients of terms that contribute little (or have redundant information) to the estimation. This approach leads to interpretable results by allowing us to clearly identify which variables had a role in detection for each month.

All statistical analyses were performed with R, version 3.2.4.

### Benchmark models

For comparison with ARGO, we included estimation results from five alternative methods. These are:

1. A seasonal autoregressive model without Google information, denoted as SAR, using a time series of the most recent 3 lags, as well as 2 seasonal lags. Specifically, the monthly time series model is comprised of time lags 1,2,3,12,24: *y*_*t*_ = *α*_1_*y*_*t*−1_ + *α*_2_*y*_*t*−2_ + *α*_3_*y*_*t*−3_ + *α*_12_*y*_*t*−12_ + *α*_24_*y*_*t*−24_ + *ϵ*_*t*_, $\epsilon_{t}∼N\left(0,\sigma^{2}\right)$. This model serves as a baseline for estimates made only using dengue time-series information.
2. Google Dengue Trends [25], which ended in August 2015. Data are obtained from https://www.google.org/flutrends/about/. Because Google Dengue Trends reported dengue intensity in a scale from 0 to 1, we dynamically rescaled it using a sliding training window to recreate case estimates.
3. A penalized multivariate linear regression model with Google Trends information only [34], denoted as GT. This is essentially ARGO without autoregressive lags, and incorporates a common *L*_1_ penalty on the Google Trends data;
4. A seasonal autoregressive model *with* Google Dengue Trends as exogenous variable, denoted as SAR+GDT. *y*_*t*_ = *α*_1_*y*_*t*−1_ + *α*_2_*y*_*t*−2_ + *α*_3_*y*_*t*−3_ + *α*_12_*y*_*t*−12_ + *α*_24_*y*_*t*−24_ + *β* log GDT_*t*_ + *ϵ*_*t*_, $\epsilon_{t}∼N\left(0,\sigma^{2}\right)$.
5. A naive method, which simply uses the case count at the previous month as the guess for the value of the current month.

All benchmark models (except the naive method) were trained by linear regression with sliding two year windows for fair comparison.

### Accuracy metrics

We used five accuracy metrics to compare model performance: root mean squared error (RMSE), mean absolute error (MAE), root mean squared percentage error (RMSPE), mean absolute percentage error (MAPE), and Pearson correlation.

Mathematically, these accuracy metrics of estimator $\hat{c}$ for target dengue case count *c* are defined as, $\text{RMSE}={\left[1/n\sum_{t=1}^{n}{\left({\hat{c}}_{t}-c_{t}\right)}^{2}\right]}^{1/2}$, $\text{MAE}=1/n\sum_{t=1}^{n}\;\left|{\hat{c}}_{t}-c_{t}\right|$, $\text{RMSPE}={\left\{1/n\sum_{t=1}^{n}\;{\left[\left({\hat{c}}_{t}-c_{t}\right)/c_{t}\right]}^{2}\right\}}^{1/2}$, $\text{MAPE}=1/n\sum_{t=1}^{n}\;|{\hat{c}}_{t}-c_{t}|/c_{t}$.

### Retrospective estimations

Retrospective out-of-sample estimates of dengue case counts were generated for each country using ARGO and the five benchmark models, assuming we only had access to information available at the time of estimation. The time windows in which we assessed the performance of our dengue estimates for each country were chosen based on the availability of official and GDT benchmark data.

These time windows are: Brazil from Mar 2006–Dec 2012, Mexico from Mar 2006–Aug 2015, Thailand from Oct 2010–Aug 2015, Singapore from Feb 2008–Aug 2015, and Taiwan from Jan 2013–Mar 2016.

## Results

**Fig 1:**
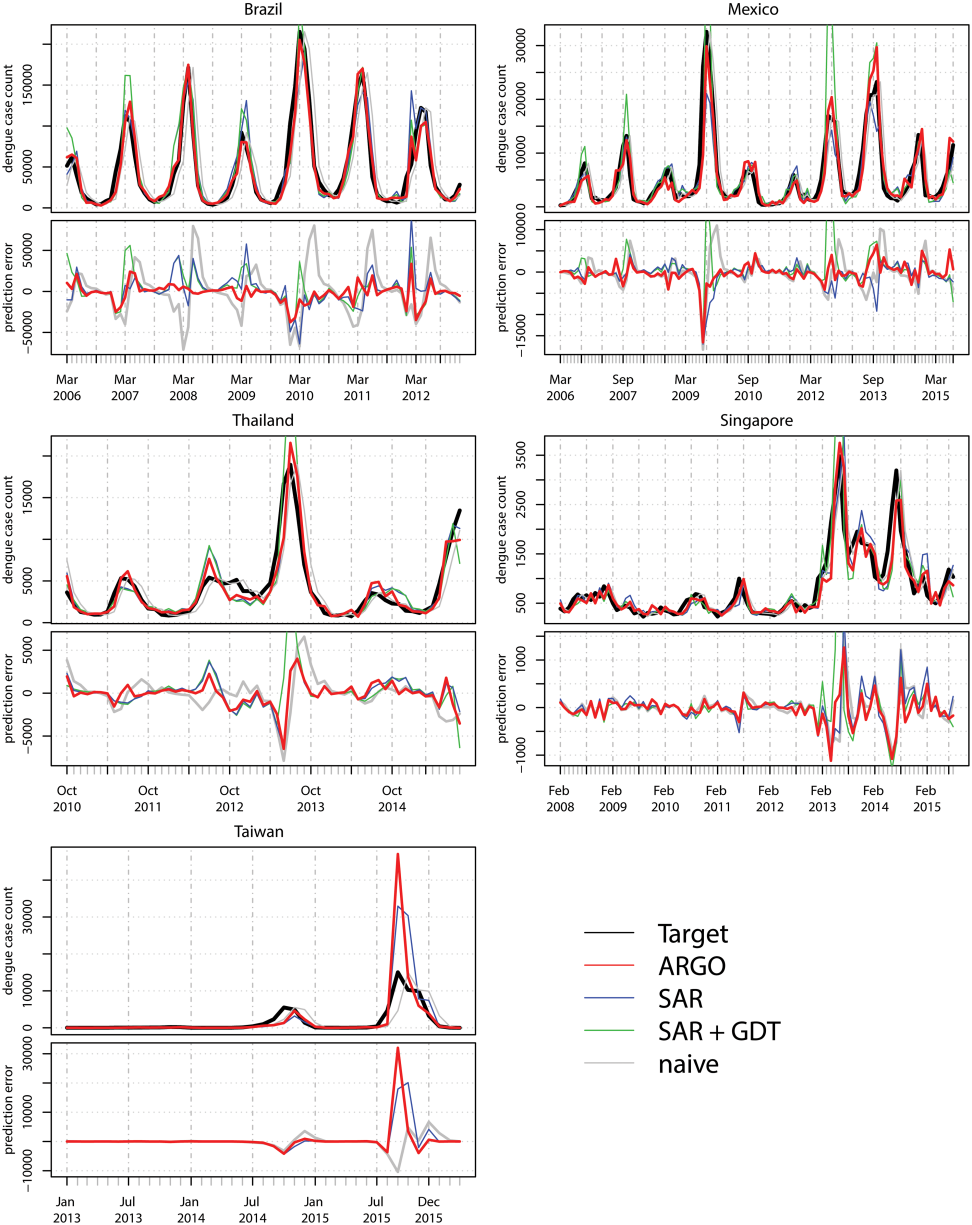
Estimation results. Monthly dengue case-count estimations are displayed for all studied countries for four different estimation methodologies: ARGO, a seasonal auto-regressive model with and without Google Dengue Trends information (SAR+GDT, and SAR, respectively), and a naive detection that estimates current month case counts using the last month’s observed cases. Historical dengue case counts, as reported by local health authorities, are shown for reference (black line), as well as the corresponding estimation errors associated to each methodology when compared to the reference.

In four of the five countries/states, Brazil, Mexico, Thailand and Singapore, ARGO outperformed all benchmark models across essentially all accuracy metrics (RMSE, MAE, RMSPE, MAPE, correlation). See Table 1. In particular, by incorporating information from the Internet searches and the dengue time-series, ARGO achieved better results than using either information alone. This accuracy improvement is reflected in the decreased errors during both peaks of dengue activity and off-season/periods with low levels of infection. See Fig 1. Unlike the seasonal autoregression with GDT model (SAR+GDT), ARGO avoided the significant overshooting problem that has been previously noted in Google Trends data ([35], [40]). This is especially notable between 2006–2008 and 2012–2014 in Mexico, and 2006–2010 in Brazil.

**Table 1:** Comparison of ARGO to benchmark models across countries and evaluation metrics. The bold face value is the best value among all methods according to each performance metric. Google Dengue Trends was not published for Taiwan and therefore the GDT benchmark is not available for Taiwan. The assessment period for the five regions, chosen based on the common available periods for all methods, are: Brazil (Mar 2006–Dec 2012), Mexico (Mar 2006–Aug 2015), Thailand (Oct 2010–Aug 2015), Singapore (Feb 2008–Aug 2015), Taiwan (Jan 2013–Mar 2016). The error value is relative to the naive, whose absolute error value is reported in the parenthesis.

	RMSE	MAE	RMSPE	MAPE	CORR
**Brazil**					
ARGO	**0.394**	**0.369**	**0.397**	**0.389**	**0.971**
GDT	0.666	0.633	0.984	0.817	0.916
GT	0.902	0.829	0.877	0.838	0.861
SAR	0.660	0.563	0.664	0.583	0.917
SAR+GDT	0.629	0.587	0.564	0.560	0.938
naive	1 (30560.436)	1 (21677.634)	1 (0.703)	1 (0.546)	0.812
**Mexico**					
ARGO	**0.680**	**0.651**	**0.558**	**0.678**	**0.924**
GDT	0.944	0.961	1.270	1.311	0.863
GT	0.950	0.927	1.097	1.100	0.861
SAR	0.790	0.737	0.776	0.815	0.911
SAR+GDT	1.249	0.986	0.779	0.854	0.891
naive	1 (3570.105)	1 (2161.018)	1 (0.816)	1 (0.492)	0.833
**Thailand**					
ARGO	**0.715**	**0.715**	**0.708**	**0.706**	**0.928**
GDT	0.880	0.868	1.494	1.284	0.884
GT	1.364	1.224	1.510	1.368	0.833
SAR	0.774	0.836	0.906	0.898	0.917
SAR+GDT	1.157	0.983	0.923	0.936	0.903
naive	1 (2058.891)	1 (1276.068)	1 (0.426)	1 (0.326)	0.852
**Singapore**					
ARGO	**0.893**	**0.889**	**0.931**	**0.917**	**0.903**
GDT	1.182	1.285	1.427	1.439	0.821
GT	1.287	1.165	1.287	1.254	0.796
SAR	1.153	1.104	1.166	1.087	0.847
SAR+GDT	2.452	1.297	1.185	1.009	0.775
naive	1 (329.318)	1 (202.651)	1 (0.283)	1 (0.230)	0.878
**Taiwan**					
ARGO	2.180	1.264	**0.233**	**0.359**	0.834
GT	12.211	4.904	1.069	0.898	0.724
SAR	1.852	1.397	0.247	0.408	**0.878**
naive	**1 (2422.559)**	**1 (1063.597)**	1 (3.248)	1 (1.601)	0.734

Taiwan shows notably different results. Of all the available models, the naive and seasonal autoregressive models have the best performance, but neither is clearly effective. The naive model has the lowest RMSE and MAE, but the worst RMSPE, MAPE and correlation, while the seasonal model shows the best RMSPE, MAPE and correlation. In comparison, the other models have a much greater RMSE to MAE ratio, indicating worse performance during high prevalence relative to the naive model. ARGO does not outperform the benchmarks in this case.

This result seems to reflect the distinct case count pattern in Taiwan compared to the other countries. Taiwan experienced little to no dengue prevalence for years until two epidemic spikes occurred in 2014 and 2015. In contrast, the other countries experience seasonal fluctuations of dengue over their entire estimation windows. This lack of predictability may be the reason that both seasonal and Google Trends-based models have greater error than the naive model, significantly over-estimating the 2015 peak for example. Yet overall, these methods show greater correlation than the naive method, perhaps because they are more responsive. Because ARGO over-estimates to a greater extent than the autoregressive methodology, this again reflects previous observations on the tendency of Google data to overshoot.

ARGO dynamically adjusts weights of dengue time-series and Google Trends data to best fit the most recent dengue behavior (See Fig. A, B, C, D, and E in S1 Text).

## Discussion

Our findings confirm that combining historical dengue incidence information with dengue-related Google search data, in a self-adjusting manner, leads to better near real-time dengue activity estimates than those obtained with previous methodologies that exploit the information separately. This also confirms that the hidden Markov model framework used by ARGO is appropriate in this context [35].

ARGO’s uniform out-performance of other benchmark methods for Mexico, Brazil, Thailand, and Singapore demonstrates its robustness and broad applicability. ARGO achieves this by balancing the influence of Internet search data, which quickly change in the face of outbreaks, and auto-regressive information, which tempers the estimations to mitigate the problem of overshooting. The application of an *L*_1_ regularization approach [34, 35, 39] helps identify the query terms most relevant to estimation at any given time, providing easy-to-interpret information as shown in the heatmaps in Figures A, B, C, D, and E in S1 Text. ARGO dynamically trains on a two-year rolling window, allowing model parameters to adjust over time to account for changes in Internet users’ behavior. The success of our methodology is based on the intuition that the more people are affected by dengue, the higher the number of dengue-related searches will be during an outbreak, and therefore the more likely Google query information will be useful at detecting dengue activity. This is observed in our findings, where the median yearly dengue case counts are strongly associated with the performance of our methodology (i.e. the higher the median yearly cases the higher the correlation of ARGO), as shown in Table 2. This is consistent with earlier findings that dengue virus prevalence is correlated with model performance in sub-regions of Mexico [38]. In addition, in Brazil, Mexico, and Thailand, the countries where our methodology works best, a clear seasonal pattern is observed in the disease incidence trends over time, as shown in Table 2.

**Table 2:** Comparison of countries/states.

*Characteristics*	Brazil	Mexico	Thailand	Singapore	Taiwan
ARGO correlation	0.971	0.924	0.928	0.903	0.834
Median yearly case count	590,000	48,000	47,000	5,400	1,700
Seasonality (correlation of SAR)	0.917	0.911	0.917	0.847	0.878
Internet penetration [41]	50%	38%	27%	74%	76%
Google market share [42]	97%	93%	99%	84%	42%
Report frequency	monthly	monthly	monthly	weekly	weekly
Population (avg. in millions) [43, 44]	198	120	67	5.2	23
Median yearly incidence (per 10,000)	29.1	4.1	8.0	10.5	6.8
Country size (10^3^ mi^2^)	3,290	758	198	0.28	14
Population density (per mi^2^) [43]	60	160	340	18,700	1,600
GDP (per capita avg. over study period) [43]	$10,100	$9,200	$5,800	$55,000	$31,900

On the other hand, the results from Taiwan illustrate the limitations of our approach. Taiwan does not present either an observable seasonal trend or a high number of dengue cases. As a result, neither ARGO nor the model using only Google search terms reliably track dengue. Low dengue-related Internet search activity during most years and sudden public interest during the outbreaks of 2014 and 2015, causing mis-calibration of the Google Trends data, may be another contributor. Other unique characteristics of the Taiwan outbreaks are that they were largely localized in South Taiwan, where *Aedes aegypti* is resident, and featured viral strains from neighboring countries rather than endemic strains [45, 46]. Also of interest is that the increased case counts occurred during periods of significantly increased temperature and rainfall [46]. The unpredictable character of these outbreaks present challenges for the performance of ARGO, and generally of all the methods considered in our comparison, but also highlight the potential of incorporating environmental predictors such as temperature and precipitation in our approaches.

While Internet penetration may seem to be an important factor in assessing the quality of Google Trends data, the statistics from Table 2 show that it alone is not as effective as dengue prevalence or seasonality in predicting the overall performance of our methodology. As an example, although Taiwan has high Internet penetration, the dengue case count may be low enough over most years that dengue-related searches motivated by other medical or educational purposes may introduce significant noise in the Google-query data. On the other hand, ARGO shows strong improvement over the seasonal autoregressive model in Brazil and Singapore, two countries with moderate to high Internet access, compared to Mexico and Thailand, which have low Internet access, suggesting that web penetration is nevertheless still an important factor. Finally, the proportion of the population within a country using Google as a search engine also provides some insight into the performance of ARGO (Table 2). ARGO shows the lowest correlation in Taiwan, which happens to have the lowest Google market share among the countries studied here [42].

Despite dengue and flu having very different biological transmission patterns, the fact that modifications to the ARGO methodology yield robust and accurate dengue estimates indicates the strength of our methodological framework. Although the monthly time scale chosen for this study was originally chosen based on data availability, inspection shows that a monthly surveillance approach is better suited for the 2-week serial interval of dengue [47].

The dengue activity estimates obtained with our methodology, like estimates from any novel digital disease detection tool, are not meant to replace dengue information obtained from traditional healthcare-based disease surveillance; instead, they can help decision-makers confirm (or deny) suspected disease trends ahead of traditional disease surveillance systems. Ultimately, the goal of this effort is to take a step closer to the development of an accurate, real-time modeling platform, where dengue case estimates can be constantly updated to provide authorities and non-governmental organizations with potentially actionable and close to real-time data on which they can make informed decisions, as well as providing travelers visiting high-risk areas with warnings. Such a platform could bring multiple information sources together, including but not limited to traditional epidemiological case reports, Google searches, crowd-sourced data, and climate and transportation information, creating a rapid response and alert system for users based on their specific location. Timely and precise detection may turn out to play a large role in reducing infections in the near future by influencing the timing of vector control efforts, hospital and clinical preparation, and providing public and individual alerts.

The platform would also enable users to verify dengue risk information with their own observations, creating a positive feedback loop that would continuously improve the accuracy of the tool. We are currently implementing two building-blocks that could help shape such a platform. The first one consists of a webpage Healthmap.org/denguetrends where dengue estimates produced with the methodology introduced in this manuscript are continuously displayed, and the second one is a crowd-sourced tool (currently in beta) that offers a user-friendly online chat system which maps dengue cases worldwide, and gives the public free access to toolkits that help reduce their risk of infection. This second effort is led by Break Dengue’s “Dengue Track” initiative www.breakdengue.org/dengue-track/. The potential impact may be far reaching, as the same models could also be used to track and map other infectious and mosquito-borne diseases, like Zika, malaria, yellow fever or Chikungunya.

Real-time implementation of our methods requires robust responses to changes in data quality, availability, and format. For example, Google correlate data shows internal variability attributed to re-sampling when the tool is accessed at different times. In addition, epidemiological data is not always published consistently by countries, creating lags in reporting that would make our methodology (which assumes having access to last month’s dengue case counts) not applicable.

In order to understand the impact of these data limitations, we performed two robustness studies of ARGO with respect to (1) the variations in Google Trends data, and (2) the availability of the most recent dengue case count data. For the first, we obtained multiple data sets containing the search frequencies of the query terms displayed on Table A in S1 Text by accessing Google Trends 10 different times during a week. We then produced Dengue activity estimates with ARGO using these 10 data sets as input. Table B in S1 Text shows that ARGO still outperforms all other methods in Brazil, Mexico, Thailand and Singapore, despite the random variations observed in Google Trends data. For the second, we retrained all the models under the assumption that the dengue case count from the past month was never available due to reporting delays. Table C in S1 Text shows that despite the unavailability of the last month dengue counts, ARGO had competitive predictive performance in the five countries/states when compared to other models (similar to the full data case), suggesting that our methodology is robust to the time delays in reporting in addition to variations in the input variables.

While our methods are designed to self-correct over time, the introduction of an intervention to curb dengue activity that could lead to a reduction in dengue cases, such as vector control or behavioral education (e.g. use of bed nets), may potentially lead our models to temporarily over-predict incidence. However, once such an intervention has been established and remains active in a given location, our models will self-correct over time to predict the new levels of dengue activity. Sporadic, nation-wide mosquito control methods would provide a bigger challenge to dengue case count predictability and, therefore, our model’s usability.

In light of ARGO’s strengths and limitations, future work should analyze the feasibility of applying our methodology to other countries, finer spatial resolutions, and temporal resolutions. This will be followed by routine reassessments of our methods to identify changes in information or potential improvements, including new search terms. As an example of such a change, Brazil has started publishing weekly dengue case counts since 2014. While our work used only the monthly resolution for fair comparison among all countries, adapting our methods to shorter time horizons for regions that provide such information would be useful.

Information on national-level dengue activity may not be ideal for decision-making at the local level since this information has been aggregated over a wide variety of potentially heterogeneous spatial environments. Future work should explore finer spatial resolution estimations to identify whether region-specific factors may improve or worsen results, similar to what has been done in [15, 38]. The five countries/states explored in this study vary on orders of magnitudes of size; for example, Brazil, Mexico, and Thailand each spans over 100 million square miles. As a result, these three countries contain wide ecological diversity and potentially varying patterns of dengue transmission among different sub-regions. It may be expected, for example, that Brazil would show different levels of seasonality in tropical compared to temperate areas. The success of finer spatial resolutions would depend on the quality of local case count and Google Trends data; the former can be affected by reporting efficiency, and the latter can be subject to Internet availability and Google use in a given region. Using national level data, on the other hand, has the advantage of smoother incidence curves for extraction and extrapolation of signal at the cost of more granular information. This is reflected in the observation that ARGO performed best in the three large countries despite the inherent heterogeneity within each country. This fits with our previous observation that a combination of higher dengue prevalence at the national level, seasonality and Google use in these countries leads to better results. We believe that these strengths and limitations also apply to extending our methodology to other countries/states besides those studied in the paper.

Producing short-term forecasts of dengue activity, in addition to the nowcast presented here should also be pursued (See [48] for such an extension for flu forecasting). Our approach may help produce dengue activity estimates in higher spatial resolutions that can lead to alert systems for people with an increased risk of exposure to the dengue virus at any given point in time. It is important to keep in mind that state-level or city-level spatial scales with low dengue activity may present similar challenges to the applicability of our approach as seen in Taiwan. The incorporation of other Internet-based data sources [48, 49] and cross-country spatial relationships should also be exploited in order to improve the accuracy in predictions.

## Supporting information

S1 Text Supporting information text. This file includes: (1) Query terms used for each country/state as Table A; (2) ARGO hyper-parameters for each country/state; (3) Aggregation from weekly data to monthly data; (4) Robustness to Google Trends variation as Table B; (5) Sensitivity to the availability of dengue case count in the past month as Table C; (6) Heatmaps of ARGO coefficients for each country/state as Figure A, B, C, D, and E.
(PDF)
