# Supplementary material for: Advances in using Internet searches to track dengue
Source: PLoS Comput Biol. 2017 Jul 20;13(7):e1005607. doi: 10.1371/journal.pcbi.1005607 (PMC5519005; doi:10.1371/journal.pcbi.1005607)

Advances in using Internet searches to track dengue

Shihao Yang[1], S. C. Kou [1,*], Fred Lu [2], John S. Brownstein[2,3], Nicholas Brooke [4]
Mauricio Santillana[2,3, *]

1 Department of Statistics, Harvard University, Cambridge, MA, USA
2 Computational Health Informatics Program, Boston Children's Hospital, Boston, MA, USA
3 Harvard Medical School, Boston, MA, USA
4 The Synergist, Brussels, Belgium

* Corresponding authors: msantill@g.harvard.edu; kou@stat.harvard.edu

Supporting Information

Table A. Query terms used for each country/state

Brazil	Mexico	Thailand	Singapore	Taiwan
dengue	dengue	โรคไข้เลือดออก	dengue	登革熱
sintomas.dengue	dengue.dengue.dengue	อาการ.โรค.ไข้เลือดออก	dengue.fever	登革熱噴藥
mosquito	el.dengue	ไข้เลือดออก	dengue.symptoms	出血性登革熱
sintomas.da.dengue	dengue.sintomas	โรค.ไข้เลือดออก	dengue.singapore	埃及斑蚊
a.dengue	sintomas.del.dengue	การ.ป้องกัน.ไข้เลือดออก	symptoms.dengue.fever	登格熱
mosquito.dengue	dengue.hemorragico	อาการ.ของ.ไข้เลือดออก	symptoms.of.dengue	防蚊液
mosquito.da.dengue	sintomas.de.dengue	สาเหตุ.ไข้เลือดออก	dengue.fever.singapore	白線斑蚊
dengue.hemorrágica	que.es.dengue	โครงการ.ไข้เลือดออก	dengue.mosquito	登革樂
sintomas.de.dengue	dengue.clasico	สถานการณ์.โรค.ไข้เลือดออก	mosquito	dengue fever
sobre.a.dengue	dengue.mosquito	สถานการณ์.ไข้เลือดออก	dengue.in.singapore	蚊子叮

ARGO hyper-parameters for each country/state

Mexico Since we found the nearest three time lags to have significant predictive effect on future dengue occurrence, we decided not to penalize these three time lags, setting $\lambda_{\alpha_j} = 0$, $j = 1, 2, 3$. We do not have knowledge of the predictive power of the later time lags, so we set a common penalty for all of them $\lambda_{\alpha_j} = \lambda_\alpha$, $j \geq 4$. We applied the same argument to the Google search terms. We further set $\lambda_\alpha = \lambda_\beta$ to reduce the number of hyper parameters, therefore $\lambda_{\alpha_k} = \lambda_{\beta_k} = 0$ for $k = 1, 2, 3$, $\lambda_{\alpha_j} = \lambda$ for $j = 4, \ldots, 12, 24$, and $\lambda_{\beta_k} = \lambda$ for $k = 4, \ldots, 10$.

Brazil We found the same pattern for Brazil as for Mexico. Thus, we set $\lambda_{\alpha_k} = \lambda_{\beta_k} = 0$ for $k = 1, 2, 3$, $\lambda_{\alpha_j} = \lambda$ for $j = 4, \ldots, 12, 24$, and $\lambda_{\beta_k} = \lambda$ for $k = 4, \ldots, 10$.

Thailand The first three time lags for Thailand were significant, but none of the Google terms by themselves were significant. This observation led us to set the hyper-parameters as $\lambda_{\alpha_j} = 0$ for $j = 1, 2, 3$, $\lambda_{\alpha_j} = \lambda$ for $j = 4, \ldots, 12, 24$, and $\lambda_{\beta_k} = \lambda$ for $k = 1, \ldots, 10$.

Singapore Singapore showed a similar pattern to Thailand, so we set $\lambda_{\alpha_j} = 0$ for $j = 1, 2, 3$, $\lambda_{\alpha_j} = \lambda$ for $j = 4, \ldots, 12, 24$, and $\lambda_{\beta_k} = \lambda$ for $k = 1, \ldots, 10$.

Taiwan The same argument applied for Taiwan, so we set $\lambda_{\alpha_j} = 0$ for $j = 1, 2, 3$, $\lambda_{\alpha_j} = \lambda$ for $j = 4, \ldots, 12, 24$, and $\lambda_{\beta_k} = \lambda$ for $k = 1, \ldots, 10$.

Aggregation from weekly data to monthly data

We aggregate the Google Trends data from weekly frequency to monthly frequency using summation. If a fraction of the week belongs to a certain month, the summing value will be that fraction multiplied by the value reported for that week.

Robustness to Google Trends variation

We include a robustness study to identify the effects of the observed variations in the (input) data acquired from the Google Trends website. For this, we downloaded 11 copies of data on different days in November 2016, and repeated the implementation of the methodology described in the main text. Our findings are presented in Table B. The mean of the 11 evaluation metric values is displayed as well as the standard deviation, in parenthesis. GDT has no variation since it is taken as exogenous in this study. If we had access to the raw data GDT is constructed from, we should expect to see similar variations as well. Autoregressive models do not suffer from these variations since they do not use Google Trends data as input. As expected, ARGO, which combines Google Trends data with time series data, suffers less from the variations of the Google Trends data than the model based on Google Trends data only.

Table B. Sensitivity to Google Trends variation. The mean evaluation metric value of the 11 different datasets is displayed in the table, as well as the the standard deviation. All values are absolute.

	RMSE	MAE	RMSPE	MAPE	CORR
**Brazil**					
ARGO	14602.591(1303.123)	9043.447(746.341)	0.329(0.029)	0.234(0.014)	0.957(0.008)
GDT	20349.593(0)	13725.535(0)	0.692(0)	0.446(0)	0.916(0)
GT	31606.088(3458.28)	20243.862(1716.244)	0.821(0.07)	0.565(0.043)	0.815(0.04)
SAR	20158.471(0)	12215.217(0)	0.467(0)	0.318(0)	0.917(0)
SAR+GDT	19220.295(0)	12732.517(0)	0.397(0)	0.306(0)	0.938(0)
naive	30560.436(0)	21677.634(0)	0.703(0)	0.546(0)	0.812(0)
**Mexico**					
ARGO	2695.046(145.838)	1532.008(79.432)	0.516(0.063)	0.355(0.025)	0.903(0.011)
GDT	3370.184(0)	2076.24(0)	1.036(0)	0.645(0)	0.863(0)
GT	4628.805(456.821)	2528.918(208.179)	1.016(0.13)	0.616(0.045)	0.705(0.065)
SAR	2821.504(0)	1593.552(0)	0.633(0)	0.401(0)	0.911(0)
SAR+GDT	4460.343(0)	2131.342(0)	0.635(0)	0.42(0)	0.891(0)
naive	3570.105(0)	2161.018(0)	0.816(0)	0.492(0)	0.833(0)
**Thailand**					
ARGO	1543.473(129.498)	911.561(43.288)	0.303(0.014)	0.23(0.008)	0.925(0.011)
GDT	1811.26(0)	1107.728(0)	0.636(0)	0.419(0)	0.884(0)
GT	2590.984(499.302)	1582.48(134.678)	0.687(0.068)	0.495(0.04)	0.82(0.05)
SAR	1592.675(0)	1066.51(0)	0.386(0)	0.293(0)	0.917(0)
SAR+GDT	2381.833(0)	1253.851(0)	0.393(0)	0.305(0)	0.903(0)
naive	2058.891(0)	1276.068(0)	0.426(0)	0.326(0)	0.852(0)
**Singapore**					
ARGO	309.492(24.395)	185.639(7.578)	0.282(0.011)	0.22(0.005)	0.895(0.014)
GDT	389.389(0)	260.421(0)	0.404(0)	0.331(0)	0.821(0)
GT	362.286(30.443)	246.596(13.725)	0.398(0.019)	0.323(0.017)	0.866(0.031)
SAR	379.794(0)	223.633(0)	0.33(0)	0.25(0)	0.847(0)
SAR+GDT	807.414(0)	262.783(0)	0.336(0)	0.232(0)	0.775(0)
naive	329.318(0)	202.651(0)	0.283(0)	0.23(0)	0.878(0)
**Taiwan**					
ARGO	2919.016(1284.247)	989.77(258.632)	0.846(0.154)	0.628(0.062)	0.873(0.026)
GT	5031.846(7248.156)	1336.656(1157.202)	4.092(1.126)	1.655(0.272)	0.848(0.062)
SAR	4487.372(0)	1485.911(0)	0.801(0)	0.653(0)	0.878(0)
naive	2422.559(0)	1063.597(0)	3.248(0)	1.601(0)	0.734(0)

Sensitivity to the availability of dengue case count in the past month

Formulation of ARGO and benchmark models assuming past month dengue case count is not available

ARGO model We now define the ARGO model as in equation (1) of main text, but we set $J = \{2, \ldots, 12\} \cup \{24\}$ assuming that the most recent month data is not yet available. We take the same $K = 10$, which includes the query search frequencies of both the current and the previous month. The slightly refined model is outlined below.

$$y_t = \mu_y + \sum_{j\in J} \alpha_j y_{t-j} + \sum_{k\in K} \beta_{k,0} X_{k,t} + \sum_{k\in K} \beta_{k,1} X_{k,t-1} + \epsilon_t, \quad \epsilon_t \overset{iid}{\sim} \mathcal{N}(0, \sigma^2), \qquad (1)$$

The same L_1 regularization is imposed to minimize the number of parameters as stated in the ARGO parameter estimation section. In a given month, the goal is to find parameters μ_y, $\boldsymbol{\alpha} = \{\alpha_j : j \in J\}$, and $\boldsymbol{\beta} = (\beta_{1,0}, ..., \beta_{10,0}, \beta_{1,1}, ..., \beta_{10,1})$ that minimize

$$\sum_t \left(y_t - \mu_y - \sum_{j\in J} \alpha_j y_{t-j} - \sum_{k=1}^{10} \beta_{k,0} X_{k,t} - \sum_{k=1}^{10} \beta_{k,1} X_{k,t-1} \right)^2 + \sum_{j\in J} \lambda_{\alpha_j} |\alpha_j| + \sum_{k=1}^{10} \lambda_{\beta_k} |\beta_{k,0}| + \sum_{k=1}^{10} \lambda_{\beta_k} |\beta_{k,1}|$$

where $\lambda_{\alpha_j}, \lambda_{\beta_k}$ are regularization hyper-parameters. For Brazil, Mexico, and Thailand, we set $\lambda_{\alpha_k} = \lambda_{\beta_k} = 0$ for $k = 2, 3$, $\lambda_{\alpha_j} = \lambda$ for $j = 4, \ldots, 12, 24$, and $\lambda_{\beta_k} = \lambda_{\beta_1} = \lambda$ for $k = 4, \ldots, 10$. For Singapore and Taiwan, we set $\lambda_{\alpha_k} = 0$ for $k = 2, 3$, $\lambda_{\alpha_j} = \lambda$ for $j = 4, \ldots, 12, 24$, and $\lambda_{\beta_k} = \lambda$ for $k = 1, \ldots, 10$.

Benchmark models For comparison with ARGO, the benchmark models also assume dengue case count of the most recent month not available:

1. A seasonal autoregressive model without Google information, denoted as SAR, using a time series of the most recent 3 months (i.e., 2 lags, because most recent month is not available), as well as 2 seasonal lags. Specifically, the monthly time series model is comprised of time lags 2,3,12,24: $y_t = \alpha_2 y_{t-2} + \alpha_3 y_{t-3} + \alpha_{12} y_{t-12} + \alpha_{24} y_{t-24} + \epsilon_t$, $\epsilon_t \sim \mathcal{N}(0, \sigma^2)$. This model serves as a baseline for estimations made only using dengue time-series information.

2. Google Dengue Trends [25], which ended in August 2015. Data are obtained from `https://www.google.org/flutrends/about/`. Because Google Dengue Trends reported dengue intensity in a scale from 0 to 1, we dynamically rescaled it using a sliding training window to recreate case estimates.

3. A penalized multivariate linear regression model with Google Trends information only [34], denoted as GT. This is essentially ARGO without autoregressive lags, and incorporates a common L_1 penalty on the Google Trends data of current month and most recent month;

4. A seasonal autoregressive model *with* Google Dengue Trends as exogenous variable, denoted as SAR+GDT.
   $y_t = \alpha_2 y_{t-2} + \alpha_3 y_{t-3} + \alpha_{12} y_{t-12} + \alpha_{24} y_{t-24} + \beta_1 \log \mathrm{GDT}_t + \beta_2 \log \mathrm{GDT}_{t-1} + \epsilon_t$,
   $\epsilon_t \sim \mathcal{N}(0, \sigma^2)$.

5. A naive method, which simply uses the case count two months ago as the estimation for the value of the current month.

All benchmark models (except the naive method) were trained by linear regression with sliding two year windows for fair comparison.

Performance comparison

As shown in Table C, ARGO has almost uniform outperformance to other benchmark models except in Taiwan. The performance of ARGO is similar to our finding in the main text, suggesting that the method is robust to the availability schedule of the ground truth data of dengue case count.

Table C. Comparison of ARGO to benchmark models assuming past month dengue case count is not available

	RMSE	MAE	RMSPE	MAPE	CORR
**Brazil**					
ARGO	**0.357**	**0.321**	**0.260**	**0.285**	**0.927**
GDT	0.419	0.372	0.370	0.364	0.897
GT	0.525	0.470	0.351	0.394	0.866
SAR	0.692	0.569	0.530	0.491	0.762
SAR+GDT	0.826	0.591	0.379	0.410	0.810
naive	1 (54101.159)	1 (40214.762)	1 (1.938)	1 (1.285)	0.423
**Mexico**					
ARGO	**0.524**	**0.536**	**0.389**	**0.527**	**0.872**
GDT	0.583	0.593	0.539	0.620	0.833
GT	0.647	0.593	0.573	0.588	0.790
SAR	0.737	0.756	0.946	0.883	0.684
SAR+GDT	2.033	1.192	0.846	0.804	0.711
naive	1 (6231.484)	1 (3900.929)	1 (2.013)	1 (1.089)	0.495
**Thailand**					
ARGO	**0.417**	**0.450**	**0.466**	**0.484**	**0.936**
GDT	0.519	0.519	0.792	0.730	0.877
GT	1.432	1.045	1.014	0.917	0.804
SAR	0.907	0.962	1.203	1.060	0.641
SAR+GDT	1.090	0.847	0.743	0.749	0.847
naive	1 (3647.191)	1 (2267.333)	1 (0.832)	1 (0.607)	0.532
**Singapore**					
ARGO	**0.748**	**0.778**	**0.790**	0.836	**0.835**
GDT	0.782	0.875	0.899	0.979	0.809
GT	1.421	0.904	0.812	**0.819**	0.765
SAR	1.432	1.433	1.590	1.407	0.491
SAR+GDT	2.634	1.264	1.144	0.964	0.621
naive	1 (513.588)	1 (312.989)	1 (0.463)	1 (0.353)	0.704
**Taiwan**					
ARGO	4.371	2.477	0.086	0.144	**0.900**
GT	30.431	13.435	0.502	0.436	0.721
SAR	2.238	1.429	**0.062**	**0.119**	0.594
naive	**1 (3691.033)**	**1 (1895.974)**	1 (21.261)	1 (8.280)	0.395

Heatmaps of ARGO coefficients

Figure A. Dynamic ARGO coefficients for Brazil.

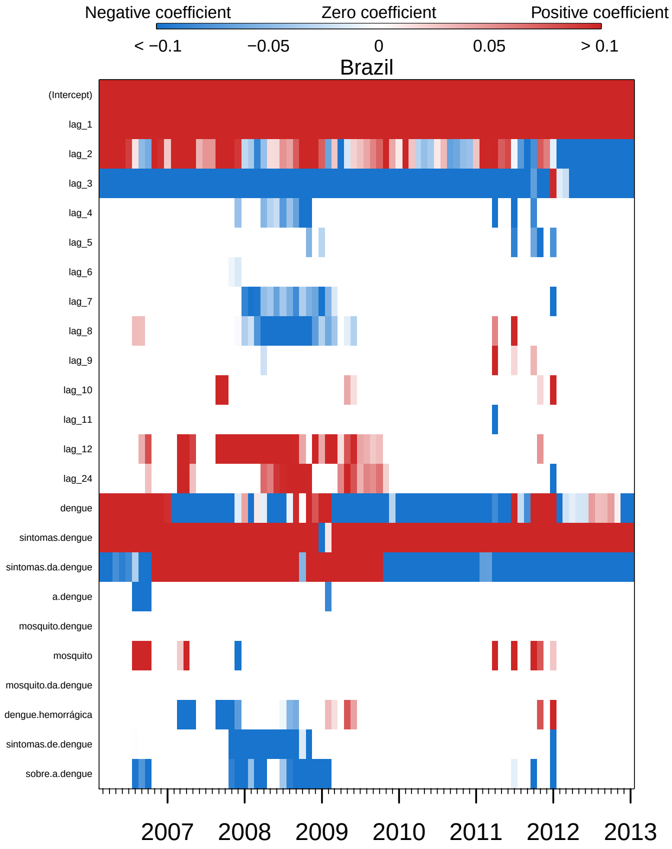

Figure B. Dynamic ARGO coefficients for Mexico.

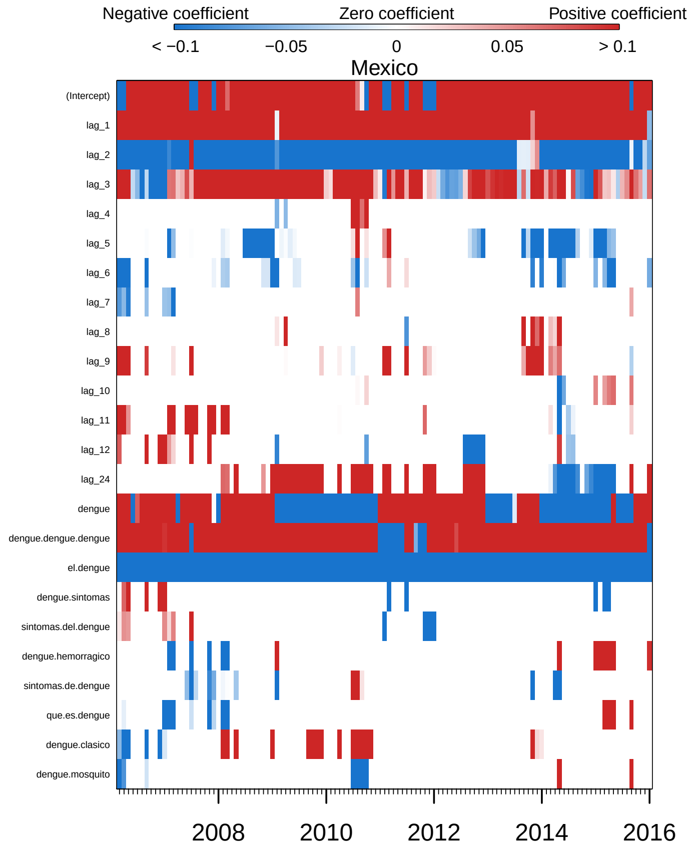

Figure C. Dynamic ARGO coefficients for Thailand. The ten query terms are listed in Table A

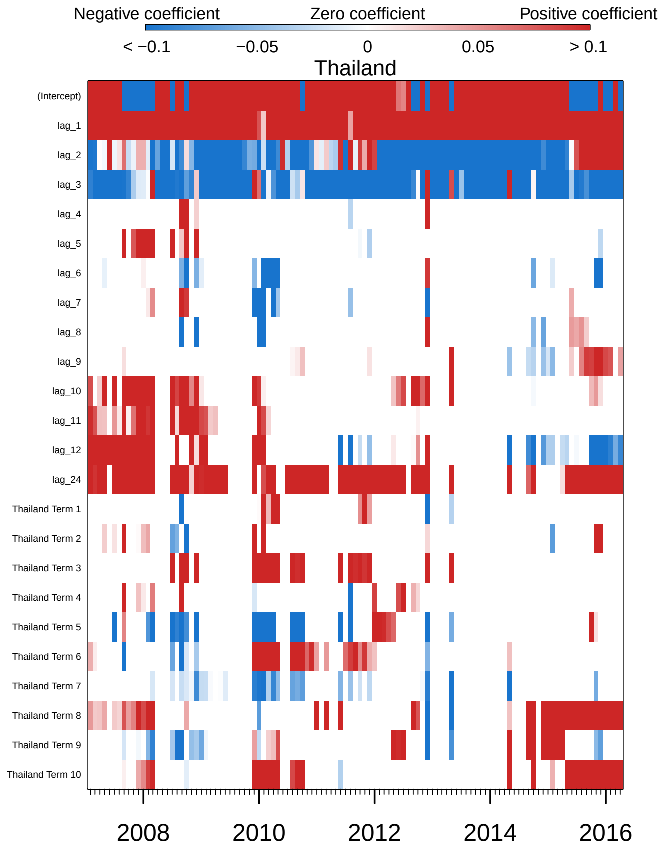

Figure D. Dynamic ARGO coefficients for Singapore.

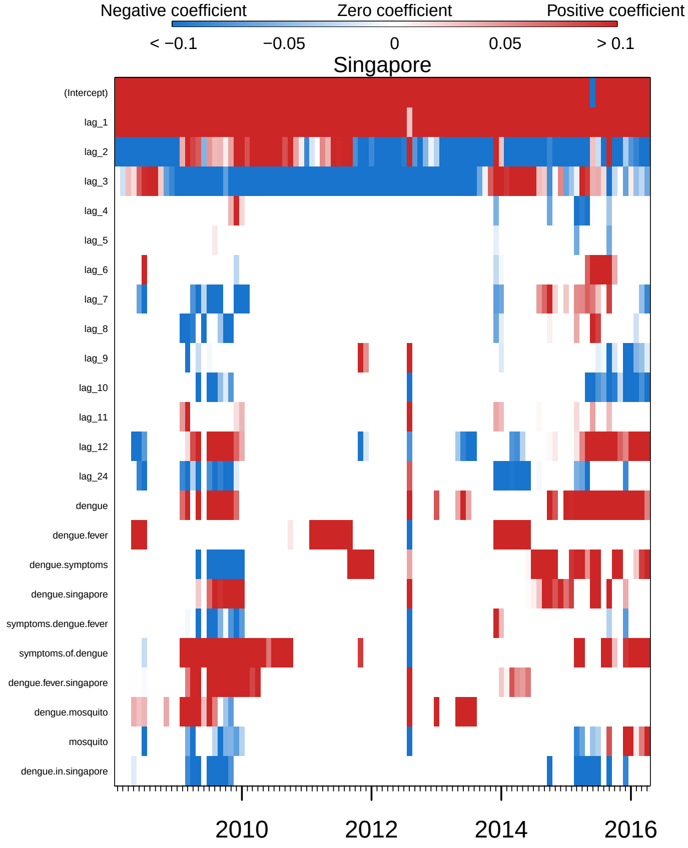

Figure E. Dynamic ARGO coefficients for Taiwan. The ten query terms are listed in Table A

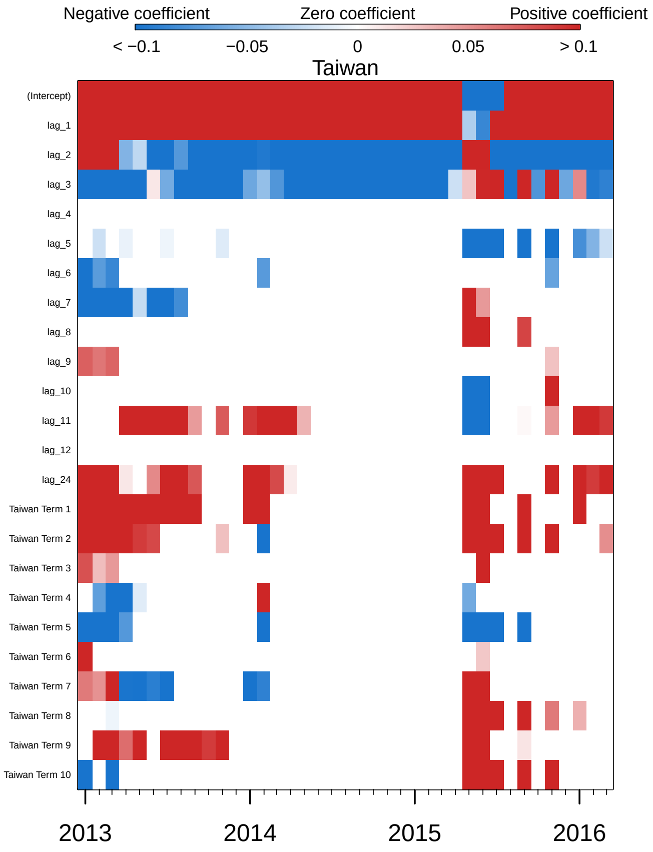

Supplement: S1 Text — This file includes: (1) Query terms used for each country/state as Table A; (2) ARGO hyper-parameters for each country/state; (3) Aggregation from weekly data to monthly data; (4) Robustness to Google Trends variation as Table B; (5) Sensitivity to the availability of dengue case count in the past month as Table C; (6) Heatmaps of ARGO coefficients for each country/state as Figure A, B, C, D, and E. (PDF) [file pcbi.1005607.s001.pdf]
